# Transition to parenthood: the needs of parents in pregnancy and early parenthood

**DOI:** 10.1186/1471-2393-8-30

**Published:** 2008-07-29

**Authors:** Toity Deave, Debbie Johnson, Jenny Ingram

**Affiliations:** 1 Centre for Child & Adolescent Health, University of the West of England Bristol, Hampton House, Cotham Hill, Bristol, UK; 2 Centre for Child & Adolescent Health, University of Bristol, Hampton House, Cotham Hill, Bristol, UK; 3 Centre for Child & Adolescent Health, University of Bristol and Bristol R&D Support Unit, University Hospitals Bristol NHS Foundation Trust, Bristol, UK

## Abstract

**Background:**

Pregnancy and the transition to parenthood are major adjustment periods within a family. Existing studies have asked parents, retrospectively, about their experience of antenatal education, mainly focusing on women. We sought to address this gap by asking first-time mothers and their partners about how they could be better supported during the antenatal period, particularly in relation to the transition to parenthood and parenting skills.

**Methods:**

Purposive sampling was used to recruit 24 nulliparous women with a range of ages from two healthcare organisations in South-West England, 20 of whom had partners. Recruitment took place antenatally at around 28 weeks gestation. Semi-structured interviews were undertaken at home in the last trimester of pregnancy and between 3–4 months postpartum. Content analysis of the interview data was undertaken.

**Results:**

Several common themes emerged from both the ante- and postnatal data, including support mechanisms, information and antenatal education, breastfeeding, practical baby-care and relationship changes. Knowledge about the transition to parenthood was poor. Women generally felt well supported, especially by female relatives and, for those who attended them, postnatal groups. This was in contrast to the men who often only had health professionals and work colleagues to turn to. The men felt very involved with their partners' pregnancy but excluded from antenatal appointments, antenatal classes and by the literature that was available. Parents had been unaware of, and surprised at, the changes in the relationship with their partners. They would have liked more information on elements of parenting and baby care, relationship changes and partners' perspectives prior to becoming parents.

**Conclusion:**

Many studies and policy documents have highlighted the paucity of parents' preparation for parenthood. This study has indicated the need for an improvement in parents' preparation for parenthood, the importance of including fathers in antenatal education and that inadequate preparation remains a concern to both women and their partners.

This paper identifies several avenues for action and further research to improve both new parents' experience of antenatal education and their preparation for parenthood.

## Background

In families, transitions represent periods of change where there are shifts in their lifestyles from one stage to another [1]. Pregnancy and the transition to parenthood is a major developmental period with important implications for parents, for the infant-parent relationship and the infant's development. Research has consistently demonstrated that it is often a stressful event and brings about more profound changes than any other developmental stage of the family life-cycle [2-4]. Women report massive changes to life-styles and routines, easy adaptation is not usual, is uniformly problematic and is not bound by any time-frame [5,6].

The present study is rooted in the view that pregnancy is an important transitional period in a new parent's life and that relevant care, information and advice is important for new parents during that period of transition. A systems theory described by Cowan and Cowan [7] depicts a five dimension continuum: a parent's anxiety about becoming a parent (the inner life), the need for a father to be more involved than his father was (the quality of relationships in the family), the demands of a job outside the home (stress outside the family), the negotiation of new roles and decisions within the family (the quality of marriage) and, because of the intricate connectedness of areas of our lives, the consequences of a change in just one area (the baby).

Many studies of the transition to parenthood have asked new parents retrospectively about their experiences and needs during their pregnancy and in the early perinatal period rather than prospectively [7-9]. However, these often include women who attend antenatal classes, therefore excluding women from more socio-economically deprived backgrounds, and focus on women not their partners [10]. Much of the earlier literature used quantitative methods and relied on the assumption that health workers understand parents' needs and experiences [10-14]. The psychological journey has been considered [15,16], experiences of pregnancy have been explored [17,18], antenatal and postnatal interventions have been evaluated [19-21] and parents' educational experiences have been reported [22-24].

The importance of the transition to parenthood on a mother's view of parenting, her parenting skills, her self-esteem and her relationship with her partner are well documented [4,15,25,26] and has been recognised at a policy level in England and Wales [27-31]. In spite of this, antenatal education continues to focus either on labour and birth and fails to address parents' needs in relation to the reality of new parenthood [14,32-35]. Women have few opportunities to gain an understanding of what to expect in the first few weeks after birth and are therefore unprepared for the demands of new motherhood [36-39]. More emotional and informational support for parents both antenatally and postnatally has been a recommendation made by several studies [8,38-40].

We are aware of no qualitative study that has asked women and their partners both antenatally, for their prospective views, and postnatally, for their retrospective views, about their educational and care needs in relation to the transition to parenthood. We hoped that by allowing the women and their partners to describe their own thoughts and views we might develop a richer account of the kinds of support, advice and information they might find helpful in becoming parents and in their parenting skills. Therefore, the aim of this study was to explore the needs of first-time parents in relation to the care, support and education during the antenatal period, particularly in relation to preparing for the transition to parenthood and their parenting skills. We also sought to identify any different issues for women who were with a stable partner compared to those without.

## Methods

Prior to the study commencing, discussions took place with local midwifery managers and supervisors. They offered their support and welcomed suggestions as to how services for women and their partners could be improved.

Information about the project was given to community midwives through professional meetings, via email from their managers and personal communication from the research midwife. Information about the project was also given to health visitor managers and permission granted to contact individual health visitors with study families on their caseloads.

### Sample

Community midwives in two healthcare provider organisations in South-West England were asked to identify all women on their caseloads who were between 18 and 35 years old, who had an uncomplicated pregnancy, who had not had a previous live baby (their own or their partners) and who understood English. The community midwives gave a study information sheet to each eligible woman when they were around 28 weeks gestation and they were asked to discuss it with their partner, if relevant and if he was not present at the time. An experienced research midwife contacted all those who gave their permission and arranged home interviews in the last trimester of pregnancy, together with the woman's partner, if she had one. Due to caseload pressures, the community midwives were unable to record the details or the number of women who did not want their contact details to be given to the research midwife.

Purposive sampling was undertaken to include parents with a range and diversity of needs and views. The intention was to recruit two groups of women with equal numbers: those with a stable partner and those without, with ten women in each group.

### Setting: models of care

The models of care that were available to the women in this study were similar, despite being spread over two different healthcare organisations and several different clinics. The pattern of antenatal care is similar in all areas and conforms with guidelines in England and Wales [31]. The woman-centred care commences around 10–12 weeks of pregnancy when they 'book' with their community midwife. Community midwives work in small groups based at health clinics but women usually see more than one midwife during their pregnancy. Women with uncomplicated pregnancies will continue mainly with midwifery care, whereas others may be placed under consultant obstetric care at the local maternity hospital. Antenatal classes, run by community midwives, are usually offered to couples from around 28 weeks of pregnancy. These generally cover issues such as labour, pain relief, breastfeeding and some aspects of early parenting. The uptake of these classes varies depending upon the area and sometimes a postnatal reunion session may be offered.

Most women give birth in one of the local maternity hospitals and return home within the first or second postnatal day. These short hospital stays mean that many new parents take their new baby home without much practical baby care instruction. On discharge, in line with postnatal guidelines [41], parents are given contact details for their community midwives. Depending on the woman's individual requirements, they are visited at home for up to two weeks. Pressures on maternity services over recent years have resulted in a reduction in home visits and many will receive a minimal number of visits at home from a midwife. A health visitor will undertake a new birth visit at home between days 10–14 days and, depending on the individual family, will discuss future contacts, whether in the home or at the clinic.

### Data collection

The research midwife interviewed each woman, and partner if present, twice between December 2005 and July 2006, firstly in the last trimester of pregnancy and secondly, at three to four months postnatally. Details about the women and men's age, employment type and ethnicity were recorded.

The semi-structured interviews were carried out using a topic guide, initially based on a review of literature and discussions within the project team. The topic guide was used to gain an understanding of the expectations and views of the women and partners who were going to be parents for the first time. It was used flexibly in response to the direction in which the women and men wanted to take the interview. Antenatal care, sources of support and information are examples of subjects covered in the antenatal interviews (Appendix). The interview guide for the postnatal interview was informed by the themes that emerged from the antenatal interviews. Postnatal interviews included topics relating to the women and their partners' support mechanisms and retrospective views of advice and information they would have found helpful in the antenatal period. Interviews were digitally recorded and transcribed; anonymity was ensured by allocating interviews unique project numbers.

For the antenatal interviews 24 women were interviewed; 20 had partners, three of whom were not present during the antenatal interview. Recruitment of women without a stable partner was slow and, due to financial constraints, we were unable to extend the recruitment process. On the assumption that there would be some who would not be available for interview postnatally, we continued to recruit women with a stable partner until more than 20 women had been recruited. By the postnatal interview, two women had moved away and two were unavailable to interview. Therefore, 20 women were re-interviewed between 3–4 months postnatally, one did not have a stable partner and one partner was not present. The interviews lasted an average of about 45 minutes (ranging from 25 minutes to one hour 20 minutes).

Permission was given to obtain data from the Child Health Data System so that the research midwife knew when each woman had delivered. The research midwife contacted each family's health visitor prior to arranging a visit to ensure that there were no adverse circumstances that might make it inappropriate to visit.

### Data analysis

Each transcript was read through and listened to several times to develop a sense of the content. The data collected were analysed manually using content analysis. Categories were established and themes were developed from these categories [42,43].

Data validation was achieved by feeding the themes back to three of the couples who attended an evening postnatal support group session. In addition, the results were fed back to the community midwives to ascertain whether they felt that the themes reflected the reality of new parenthood in a wider context.

### Ethical considerations

Approval was acquired from the appropriate NHS Trust and University Ethics Committees and research governance issues were approved by the two relevant Healthcare Trusts. Before the interview the research midwife discussed the study, assured confidentiality and ensured that the women and men understood that they could withdraw from the study at any time with no adverse effects on the care that they would receive. She also asked permission to record the interview and a consent form was signed.

## Results

The women who were interviewed had a mean age of 25.9 years, ranging from 18 to 35 years old. The partners varied in age from 19 to 37 years old with a mean age of 27.9 years. The women and their partners were mainly White-British but there was one South-East Asian couple, and three where one partner was White-British and the other Brazilian, Dutch or Asian. They had a range of socio-economic backgrounds and came from several different geographical areas of the two healthcare organisations. The women's employment status was varied, for example, these included several different professions (n = 6), administrators (n = 4), teachers (n = 2), secretaries (n = 2), looking after the home (n = 2) and manual worker (n = 1). There were also two women who were unemployed and one who was a student. The partners' employments ranged from those who were manual workers (n = 7) to those who were professionals (n = 6). One was also a student, one who was unemployed and another who was on Incapacity Benefit.

We aimed to recruit equal numbers of women who were in a stable relationship and those who said that they were not. However, recruitment of women who were without a partner was very slow and we recruited only four in the time available. Nevertheless, we found their views were similar to other women in the study and they did not introduce subjects that had not already been raised.

### Themes arising from the interviews

The themes that emerged from the antenatal interviews included: types of support received and available to the women and their partners; their views on their preparation for parenthood, the postnatal period and baby care; and the information they received and sources of this information. Postnatally, issues included some of those above (support, information and preparation) but in addition, the following were highlighted: breastfeeding and the pressure to do so; parents' relationships and the challenges they had been, and were, going through; and partners' perspectives on their involvement and inclusion in the care his partner had received both antenatally and postnatally. Parents also expressed feelings such as fear, excitement and joy about becoming parents. These themes will be discussed below, following the transitional process from pregnancy to parenthood, rather than the order of importance.

### Support received and available

The women identified five groups of people who were important to them for support: their partners, their own parents, friends and colleagues, health professionals and antenatal and/or postnatal groups. The partners, however, only mentioned their own partner, their colleagues and health professionals as avenues of support that were open to them.

#### Parents

The main area of support that the women expected to, and felt secure in turning to, were female relatives, mainly their mother, but aunts, grandmothers and sisters were also mentioned.

*My mum...she's been pretty good. She knows not to overstep the line. She's suggesting but she won't ever say*.

[mother #20045, postnatal interview]

The importance of the woman's mother really stood out. This was in contrast to the men who occasionally mentioned their own father, mostly in the sense that they wanted to offer their own child a different fathering experience.

Postnatally, the practical aspect of support and help that their mother gave them ranged from aspects of baby care to housework, cooking and/or babysitting, so that the couple could have 'time out'.

*Everything *[things her mum helped her with]. *Draw a bottle, tea, cooking, housework, everything*.

[mother #20037, postnatal interview]

#### Friends and colleagues

The women valued friends who had been through childbirth recently or who had experience of childcare. 'The information and support they could offer was greatly appreciated and sought after. In some instances these were relatively new friends who they had met at their antenatal classes but who had had their babies before them. The sharing of experiences was of paramount importance, in one instance this included the helpfulness of an interactive website. In comparison, the men appeared to lack support networks, some even felt that they had no-one to turn to, apart from their partner and, for some, their work colleagues.

*Yes, my colleague's just had a baby, about 6 months old now ...then we've got the old administrator who says what it was like in her day and things like that what the pregnancy was like and the involvement of a man*.

[partner #20010, antenatal interview]

Others felt they could only turn to health professionals, due to a lack of support from other sources. One father felt very strongly that, because he was employed, he was unable to access any support and therefore felt punished for working.

*...I mean all my sort of friends from school I have lost contact with and I haven't really got any friends, nothing like that, it's basically work. I felt that, because I worked, I wasn't being allowed to be involved in any of it. I felt punished for working...you are missing everything*.

[partner #20052, postnatal interview]

#### Health professionals

The support received from community health professionals was described as being quite varied but generally more positive than negative. Comments related to the importance of continuity of care, both from the midwife and health visitor, especially because this was often not achieved. The women also thought that the role of the midwife in the postnatal period should be longer than two weeks due to the trust that had built up antenatally.

*....in those two weeks you've built up such a relationship. I felt comfortable and... a few weeks is not long enough for breastfeeding and all that...When she left I felt very alone.... Because she takes all my notes and I didn't have any contact numbers for the health visitor. What do I do in the next week?.... A relationship with someone, it's trust isn't it*? [mother #20058, postnatal interview]

Those families who had received only one home visit from the health visitor, in contrast to frequent midwives' visits, were critical of the small amount of contact.

*She just came the once *[health visitor]*....she said we don't visit anymore. You will have to come up to the surgery*.

[mother #20039, postnatal interview]

#### Antenatal and postnatal groups

Some of the women had attended antenatal classes. These classes are usually held during the day, which makes it difficult for some working men to attend with their partners. Women who kept in postnatal contact with those in their antenatal group greatly valued the support. One or both parents spoke highly of this source of support and mentioned it several times.

*....the people who are really useful are the people that go through it at the same time...... Without that we'd really be struggling, without that network of mums that H *[his partner] *only met through antenatal classes....we don't need any help/support, or very little, other than this antenatal group*.

[partner #20058, postnatal interview]

The usefulness of this 'support' was mainly perceived as reassurance; that, as new parents, they were all going through similar difficulties and experiences. In addition, it was encouraging that trying out different solutions to problems to find out what suited them best was not only normal and acceptable but also common practice.

*Yeah we all get together and sort of lash things out and talk, babies...and you know everybody learns from their own experiences and if we all can sort of contribute, that's really good*.

[mother #20055, postnatal interview]

It also provided a forum in which the mothers (since they often consist only of women) could exchange hints and tips as to what had 'worked' for each of them, eg. in relation to sleeping problems. The mothers often then fed the advice and reassurance back to their partners.

### Preparation for parenting and baby care

Both parents alluded to the fact that antenatal classes were mainly aimed at the woman and they were generally not well attended by expectant fathers. They said that the topics covered in the classes were useful but mainly concentrated on pregnancy and birth, with little if any, discussion about parenthood.

*I would say that that was one thing that I haven't received the information on, the baby element, bay care...when it is here and that's probably why I'm not so confident really*.

[woman #21004, antenatal interview]

Antenatally, although the expectant parents felt excited about life with a baby they also expressed feelings of apprehension about caring for their baby about both the practical and general aspects of caring for a baby. They felt unprepared for becoming parents. The men articulated feelings of ignorance and fear about soon having a baby at home.

*...how to hold a baby. I know the principle, but the actual doing it....The fact that you can't communicate, you can't talk*... [partner #20010, antenatal interview]

Postnatally, in the first few weeks with their new baby, many parents mentioned feelings of surprise, confusion and excitement. They also spoke of overwhelming emotions towards their baby: amazement, love and a sense of great responsibility; that it was a life-changing event.

*Every time I see her I still cannot believe she is mine or I am really a mum now (laughter)....It changes your whole life completely...there are no words to describe it really, but it is just overflowing with joy*.

[mother #20036, postnatal interview]

The parents drew attention to the different ways in which they each interacted with their baby and the consequent reactions their baby displayed. For example, parents noticed that the father often played more with their babies, especially on return from work but that the mother was often able to calm their baby more easily when upset.

*And sometimes my husband is quite jealous because every time she sees me, it is like, she sees me as comfort you know. I am the one feeding her, singing to her something like that. Every time she sees her dad she is always playing. My husband says, how come she does not sleep with me, every time she sees me it is like playtime. She always plays with her dad*.

[mother #20033, postnatal interview]

### Type and sources of useful information

Parents gathered, and were given, information from a variety of sources, both before and after their baby was born. Family and friends, work colleagues, healthcare professionals, discussions at antenatal appointments and antenatal classes, leaflets and books, television, videos and the internet were all referred to. In addition, as previously mentioned, their own parents' knowledge often appeared to be valued even though it was not usually recent.

The midwife was generally seen as a reliable source of information and someone to whom the woman could turn for advice and support. The two main sources of information given out by midwives were the NHS Pregnancy Book, a Department of Health, evidence-based, comprehensive guide given to all expectant parents [44], supported by a variety of leaflets. The Pregnancy Book was singled out by many parents as being very useful, comprehensive and something that they referred to on a regular basis.

*Yeah, it's brilliant *[NHS Pregnancy Book], *I always look there when I need reassurance or just a little read through really*.

[woman #20004, antenatal interview]

Most parents had read books, some of which they had bought, others had been given or lent them by family and friends, or borrowed from the library. Antenatally, parents watched television programmes relating to pregnancy and birth, and accessed the internet. Commercial gift packs, given to women early in pregnancy, were mentioned by many women, especially those who had signed up to receive weekly emails about their pregnancy. However, such advertising was not acknowledged as potentially influential in their choice of products. Nonetheless, whether in the form of literature, television programmes or services, there appeared to be few resources intended for fathers either ante- or postnatally, especially for those who were unable to take time off during the day.

*I was sort of trying to push for information and I was finding it hard to get...from a dad's point of view*.

[partner #20014, antenatal interview]

*For blokes that don't work the same sort of hours as I do there is actually one *[a group] *run with just dads*, *run just through the day...but I mean there is nothing I heard of for dad's in the evenings....I would have loved to have done it. You know, meet a couple of people*. [partner #20052, postnatal interview]

In discussing what else might have been helpful to them, eight parents raised the idea of a new DVD that could be specifically targeted at them. For those other parents who themselves did not mention the idea of a DVD, this was introduced at the end of the interview. They felt that an innovative DVD would be a helpful and supportive resource to have when entering parenthood. They thought that if one was available, late pregnancy would be a good time to watch it when they would have time to do so.

*It would be nice if the midwives could do a mini-video like thing? About mothers' experiences? Let other mothers know about what's going on, what's going to happen to them....Yeah, 'cos you could sit in your own comfort of your own home and watch it in detail, and you can always play it back to yourself*.

[woman #20007, antenatal interview]

*I've looked in DVD stores and found that there isn't really that much, it's more to do with exercise and pregnancy, but that would be an excellent addition*.

[partner #20024, antenatal interview]

### Breastfeeding

Women mainly gained advice and information about breastfeeding from midwives, either in antenatal classes where videos might have been used, or through being given leaflets to read.

*but we did one yesterday on breastfeeding ....the breastfeeding was one of the most informative for me.. they picked up in the itinerary that this one was gonna be about breastfeeding so none of the men that normally come came*.

[woman #21004, antenatal interview]

Men often excluded themselves from the antenatal breastfeeding sessions because they did not feel that breastfeeding was relevant to them. Their partners were usually of the same opinion and this was sometimes backed up by midwives who told them they didn't need to attend when breastfeeding was being discussed.

*I think if it was general feeding he would have, because it was just breastfeeding he didn't feel that he'd have anything to do with it*. [woman #20022, antenatal interview]

The majority of women started breastfeeding and wanted to succeed in doing so but many commented on the pressure they felt, from health professionals, to continue.

*We struggled on for six days trying to breastfeed. It wasn't so much that the midwife pushed you to carry on breastfeeding, because when we made the decision that we wanted to stop they were actually really supportive. I was worried that I was going to get given a hard time, but they were actually fantastic*. [mother #20055, postnatal interview]

### Parents' relationships

During the antenatal interviews the women and their partners were aware that they themselves might change when they became parents but made few allusions to the possibility of any change in their relationship.

*The bit afterwards I think we both know that we'll change quite a lot, personality wise and stuff, I think we'll both change a lot*. [woman #21003, antenatal interview]

This was in contrast to postnatally when parents talked openly about the additional stresses on their relationships. They expressed surprise at the demands that had been placed on their relationships and the effect that having a baby had had on them as a couple.

*We don't argue, we don't snap at one another. And......knowing I was doing it...for no good reason and was upsetting her ... things would've been different if we hadn't been as strong together. If you got any, any stress in your marriage, and a kid, they would struggle I think*. [partner #20051, postnatal interview]

There was also some sadness and bemusement that no-one had talked to them about the changes they would experience in their relationships. Postnatally, they could understand why the changes had taken place but would have preferred to have been warned in advance.

[Partner]: *I mean if the awareness could have been made a lot more, because no one ever really spoke to us about that other side...and the relationship with us and the baby. It was never the relationship with us*.

[Mother]: *About relationships, like, it was always about the baby*. [postnatal interview #20052]

Making time to talk and spending time together were recognised as valuable ways to reduce relationship tensions. Although these were common feelings, the parents made many positive comments about feeling like a family and enjoying their baby.

*I think that we're probably closer if anything because we sort of feel like a complete package*.

[mother #20036, postnatal interview]

The interview experience itself allowed couples 'time out' to discuss their feelings, concerns and relationships. It often proved to be a positive experience that highlighted the need for them to make time to communicate their feelings. For one couple, the partner implied that they might have split up if they hadn't realised the need to make that time.

*If this carried on we would not have been together for this meeting. We sort of sat down and we tried about two or three different ways and thought about it .. now we've got just back to the way we were before. The only difference is we've got a little girl*. [partner #20051, postnatal interview]

### Partners' perspectives

The womens' partners generally felt very involved with the woman's pregnancy but often felt excluded from antenatal appointments and classes.

*I didn't find it very useful to be honest with you, because it was not ... it was more on S*. [his partner] *and her pain. I don't ... I wasn't ready and they didn't involve ...The only thing they said I could really do was just be there and that was it really*.

[partner #20013, antenatal interview]

The men mentioned there was a lack of information for them and that they were personally given none of the contact numbers for midwives, health visitors etc.

*I was terrified... you know the sort of the care is, it is very much geared towards the women, I am not aware of anyone or have any numbers that I can speak to*.

[partner #20058, postnatal interview]

They generally felt unprepared for caring for a baby, both the practical aspects as well as concern about being unable to communicate with their new baby.

*I' m absolutely petrified..suddenly they are going to release you with this child that doesn't communicate in English and you're going to take this thing home*.

[partner #20023, antenatal interview]

Some also reflected on how difficult it had been to go back to work and to achieve a work-life balance. They also spoke about feeling excluded from advice and support once they were working.

*...trying to get a balance between the relationship and the baby. After two weeks I had to go back to work but I did not want to leave her*. [partner #20039, postnatal interview]

## Discussion

This was an exploratory study to identify the needs of first-time parents in their transition to parenthood, with the intention of enhancing the education and support provided for them. It was embedded in the notion that becoming a parent forms a major transitional period in a new parent's life. It provides an empirical example of a theoretical framework of systems that was described by Cowan and Cowan [7]. This highlights the interconnectedness of the different elements in our lives and that a change in one area, for example, a new baby, affects all other areas. Like previous studies, it demonstrates that new parents continue to feel unaware of, and unprepared for, the transition in relationships [14,39]. However, it also provides new insights into how ill-equipped new parents are for parenthood: both the practical aspects of caring for a baby and the change in themselves on becoming parents. This process of change is not bound within a particular time-frame. It may start during the antenatal period but the feeling of, 'becoming a parent', may occur as early as in the first month or it may take up to 12 months after birth [6]. Whilst these results have relevance for local maternity services, it is likely that they may have relevance for maternity services and midwifery education worldwide.

### Strengths and limitations

No claims are made as to the wider transferability of the findings of this study, although it is hoped that the transparency of the method of analysis helps to establish the credibility and trustworthiness of the findings. The parents taking part were purposively recruited, they varied in age and were from a variety of different socio-demographic backgrounds and geographical areas across a city in South-West England. We were only able to recruit a small number of single parents but we found that their views did not differ from women who had a partner. The study excluded parents who did not have English as their first language. The views of the parents included in this study, who are predominantly British-born, may differ from those in black and minority ethnic groups due to differing practical and cultural needs. It is possible that the inclusion of a broader, more heterogeneous sample of parents would lead to further development and widening of the themes. Further studies, specifically working with other ethnic groups, are necessary to discover the similarities and differences in the issues that they might raise. The numbers of parents recruited to this study was small, therefore we were not able to look at differences in age in relation to the specific needs that they identified.

Participant validation was ascertained by feeding back the findings to three of the couples who attended an evening postnatal support group session. The results have also been fed back to community midwives who felt that the aspects highlighted by the parents reflected the reality of the experiences of new parents in a wider context. These elements confirm that the systems theory of transition underpinning this study is understood and has relevance to others, thus verifying its credibility.

The aim of this project was to gain a better understanding of possible improvements in the care, support and education that parents receive whilst they are adapting to parenthood. Twenty-four women and twenty of their partners were recruited and interviewed antenatally. Twenty of these women and eighteen partners were interviewed postnatally. Whilst some issues they raised are supported by earlier studies [14-16], other topics they highlighted provide new insights into the continuing issue of new parents who are unprepared for the transition to parenthood. In spite of using a range of resources to help prepare themselves for the birth of their baby, most parents felt they had been inadequately prepared for the reality of parenthood. This is in spite of recommendations made by several studies and recent national guidelines that more emotional and informational support should be provided for parents, in the antenatal and postnatal periods [8,9], [22-24,31,41]. During both sets of interviews, the practical aspects of caring for their baby in a frank and touching manner were raised as real concerns, especially by the men. Unlike previous studies, the parents in our study were asked both antenatally and postnatally about their needs in relation to the transition to parenthood [7-9]. It is problematic to ask parents postnatally about the information they would have liked in the antenatal period because of the influence of their experience of early parenthood.

Examples from countries across the world have highlighted the importance of involving fathers, providing them with information and preparing them for the changes in their relationships with their partners [9,11,14,18]. Although men are more involved in the antenatal care of their partners than in the past, our study suggests they felt frustrated by the lack of inclusion, involvement and information for new fathers. In particular, they were made to feel that breastfeeding was nothing to do with them, despite the recognised importance of the role of partner support [45]. Even though recent research has indicated that fathers contribute to child development [46,47] the men in our study felt that their emotional and psychological needs were neglected, that they were excluded from discussions and that they were unprepared for fatherhood.

Postnatally, both parents highlighted the importance of being prepared for changes in their relationships. Even though changes in couples' relationships post-birth have been well-documented [25,26] most parents were unprepared for and shocked by the stress placed on, and the decline in, their relationships. In general, they understood why it was happening but few had taken the time to sit down and talk to each other. This strain can be serious and even result in relationships breaking up [3].

The parents in our study described elements of the transitional process that was important to them, both prospectively and retrospectively, and emphasised the importance of adequate preparation beginning in the late antenatal period. They identified the need for an educational DVD; the suggested content of which was broad-reaching and included practical advice about caring for a baby and issues to do with relationship changes. The DVD does not exist as yet but they were particularly enthusiastic about it because they felt it was something they could watch together, alone, with other expectant parents, view just specific sections and do so at a time and place that was convenient to them. This flexibility was really important to them. Funding to develop an educational DVD and evaluate its impact is currently being sought.

## Conclusion

This study has highlighted the paucity of parents' preparation for parenthood, the lack of inclusion of fathers from antenatal education and that the inadequate preparation remains a concern to both women and their partners. This is in spite of many studies and policy documents highlighting the importance of these issues. Our findings are both relevant to healthcare providers and researchers and can be used to improve antenatal education. Examples where midwives and other childcare educators can influence current and future antenatal education include: inviting new parents to antenatal classes to discuss their recent experiences of becoming parents; sending a written invitation to both parents, by name if possible, to antenatal classes; encouraging partners to attend the session on breastfeeding; rewriting education materials so that they are more inclusive of the women's partners; increase future parents' awareness of relationship changes and actively promote parents to discuss potential changes.

Future research in antenatal provision should, firstly, examine the effectiveness of methods by which additional information and support can be offered to new parents whilst not imposing additional work on healthcare professionals. This could include encouraging new parents to attend antenatal classes to talk about their recent experiences. Secondly, further research is needed to explore the role of fathers in antenatal education. Maternal input has been well documented, but little is known about what types of interventions would be effective in improving the experience of fatherhood in the early postnatal period. Thirdly, this study should be repeated with first-time parents from black and minority ethnic groups and in other healthcare systems to provide a basis for understanding the needs of new parents from a broader international perspective.

## Competing interests

The authors declare that they have no competing interests.

## Authors' contributions

TD formulated the study design, carried out the data analysis and wrote the manuscript. DJ participated in the study design, data collection, data analysis and commented on the draft manuscript. JI contributed to the design, and commented on the draft manuscript. All authors read and approved the final manuscript.

## Appendix

Antenatal interview schedule

If both future parents present: pay attention to the individual parent's responses and that both are included.

For this interview I would like you to try to think past the birth of your baby, if possible, to the time when you will be parents.

**Can you tell me about your family? **How many there are, how close, number of siblings etc.

Have you any prior experience with babies – family's/sibling's/friends' children etc?

Which parent – both or just one? Find out how long ago, how much contact did they have, how much experience did they gain? Are they still in contact?

Have you any friends/family with babies with whom you are in close contact?

How long ago, are the babies/children still young? How much 'looking after' of the babies did they do?

Do you feel that the contact you have had with your friends'/family's babies/children will help at all with becoming parents or being parents?

**(Both parents) **In what way? Anything in particular?

Have you friends/family to whom you can both/turn to for advice, support and/or information about being pregnant, delivery and/or becoming parents?

Have they found it helpful? Do they think it will be helpful? Any particular aspect(s)?

What do you think you want and/or need from health care professionals during your pregnancy, especially in relation to becoming parents and gaining parenting skills? Do you think they may be helpful? In what way?

Do you have any expectations about the information/education/preparation you might be offered/given by health care professionals?

What sort, when, who from, do you think it will it be useful?

Would the partner/father like to be present at AN appointments, AN sessions, etc? Has he been included so far?

Would the mother/partner like her partner to be present? Does she feel that he has been included so far?

*If not*, is it because the appointments etc have been in the evening, or what is the reason?

Have you thought at all about parenthood, how you might adapt to it? 

Are they excited/anxious? Made any provisions for support, etc?

Ideally do you know what sort of input you would like during your pregnancy?

What would this be like, who from, concentrating on what particular aspects?

Have you received any information about your pregnancy/being pregnant, giving birth or parenting yet?

*If yes*, at what stage, where/who from? Was it requested or was it offered? Was it helpful?

*If no*, would they have liked to have received anything? Any idea at what/from which point, and in what format?

Have you read anything about pregnancy, giving birth or parenting yet?

*If yes*, where/who from? Was it helpful? Was it enough, would they like any more, what other sorts of things would they like to find out about?

*If no*, is it that they think that they will be told all that they will need to know?

Or are they going to read something, or are they happy not reading anything/finding out about the antenatal stage, giving birth, becoming parents?

**Did you plan to become pregnant this time? **(NB. Difference between planned, wanted etc). How long for, any assistance, both parents in agreement?

How many weeks pregnant were you when you first found out you were pregnant?

Is there anything that you would like to mention/ask that you haven't been able to already?

We would like to thank you very much for your time and, if it is still ok with you, we will contact you after your baby is born and interview you both again when your baby is 3–4 months old.

## Pre-publication history

The pre-publication history for this paper can be accessed here:


